# Supplementary material for: Flow signal change in polyps after anti-vascular endothelial growth factor therapy
Source: PLoS One. 2020 Oct 23;15(10):e0241230. doi: 10.1371/journal.pone.0241230 (PMC7584188; doi:10.1371/journal.pone.0241230)
Supplement: Attachment — Submitted filename: Response to Reviewers.docx [file pone.0241230.s001.docx]

Dear Editors-in-chief:

We appreciate the reviewers’ valuable questions and the chance to improve our manuscript. The concerns on the methods (e.g., en Face vs. horizontal scan, exclusion criteria..), figure 1, table and text had been explained and revised accordingly. We hoped after these revisions, the Plos One will consider our paper interesting and deserve to be published.

Best regards,

Shih-Jen Chen MD, PhD

| Comments from reviewers | Response | Changes made in the manuscript |
| --- | --- | --- |
| Reviewer 1 |  |  |
| 1. Kindly explain why horizontal OCTA scans have been preferred over En-face OCTA scan | The En-face OCTA and horizontal OCTA scans were both used for flow signal detection. The signal of En- face OCTA was studied for flow signal at areas of polyps and BVN shown in ICGA. To confirm the flow signal, especially in equivocal signal after anti-VEGF, horizontal OCTA scan were used across the polyps line by line for confirmation. This is why we set the definition of positive flow signal in 3 consecutive horizontal scans on the polyps. | This was further clarified in Page 5 line 108 |
| 2. Presence of Branching vascular network (BVN) complex has not been recorded. Any correlation between polyps and BVN complex could also have been identified | Thank you for the constructive advice. We had evaluated the presence of BVN in OCTA and ICGA, and found that 9 among 13 patients had BVN before the treatment. The size of BVN ranged from 0.75 to 3.0 disc area(DA) (average of 1.42±0.75 DA). After treatment, the size of the BVN remained unchanged in this short term 3 months follow up. This was in accordance with previous studies that the BVN had poorer response to anti-VEGF than the polyps did. | This was further clarified in Table 1  Page 8 line 159  Page 9 line 167  Page 11 line 225 |
| 3. Fig 1 (F, G, H) showing En-face OCTA of low flow polyp, could not be considered a proper scan. The manual segmentation is not at the appropriate level to be able to visualise the polyp. | Since the case had prominent SRF, it was hard to visualize the polyp on the En-face OCTA even with manual segmentation. Fig 1 (F, G, H) explained the definition of low flow polyps in our study, of which no flow signal in 3 consecutive horizontal OCTA scan in the corresponding polyp location of the ICGA. There was an adjacent high flow polyp pointed out by arrow for comparison. | New figure 1 (revised F, G, H) would be submitted, and figure legends had been revised in Page 6 line 129. |
| 4. Figure 2 is incomplete. No details of high flow polyps given. | Figure 2 is the flow chart of all the outcomes of polyps. The blanks might be due to format converting error and was probably incompletely uploaded. We will re-submit and upload again and will check with the editor office to ensure the completeness of the figure. | Figure 2 would be re-submitted. |
| 5. Figure 3 depicting ICGA after treatment, shows complete remission of polyp 1 and 3. However, flow signals in the polyps still persist on OCTA after treatment. How can it be explained? | This is a very interesting point. We did see 2 among 8 polyps had completely regressed in ICGA without fluorescence after treatment yet still had flow signal in OCTA. Theoretically, the OCTA scan could detect very slow flow signal with velocity of 0.2 to 3 mm/s. Therefore, though the ICGA after the treatment revealed complete regression of the polyp, there might be slow flow remaining in the area that could be defected by the OCTA but not visualized in ICGA. | This was further clarified in Page 13 line 258 |
| 6. A sample of 2 low flow polyps is clearly inadequate to conclude the relevance or to predict clinical response to anti-VEGF | Thank you and we agreed with you that this is the limitation of our study with small numbers of cases. However, on the other hand, because of the wide detection of flow signal by OCTA (theoretically 0.2 to 3mm/second), the low flow polyps may indeed the polyps with very low filling flow of the polyps and the number may be quite few in symptomatic patients with PCV. It will be interesting to see if there are more low flow polyps in asymptomatic patients with PCV. | This was further clarified in Page 13 line 265 |
| Reviewer 2 |  |  |
| 1. PCV eyes with poor visual acuity may not be able to fixate well during OCTA scan leading to motion artefacts. Were there any eyes excluded from the study and what were the reason for their exclusion? I believe providing details on the exclusion criteria and the number of patients that were excluded from the consecutive recruitment process is very important as it conveys to the readers how practical it would be to examine PCV eyes with OCTA and under which circumstances the examination of flow within polypoidal lesions would be feasible. | The visual acuity of our naïve patients in this study ranged from 6/30~6/7.5. Patients with poor image quality due to poor fixation even with good visual acuity were exclude from the study. During this study period, there were 49 patients who met the exclusion criteria and were not enrolled in this study. These exclusion criteria included history of previous treatment with IVI or PDT, poor image quality due to fixation loss, medium opacity, massive hemorrhagic PED or submacular hemorrhage obscuring the polyps. | The exclusion criteria had been revised in line 81 and line 134 |
| 2. The authors defined high flow lesions as those that with the presence of polyps on both ICGA and OCTA as determined by the examiners, on the other hand, low flow polyps were only detected on ICGA.  Were the examiners for ICGA and OCTA blinded from each other? How many image graders were there? | There were 2 retinal specialists to interpret each color, OCT and ICGA images separately. They were blind to each other’s results. Lesions were enrolled only when they were considered to be polyps by both retinal specialists. The en face OCTA and manual segmentation of each polyp was then studied by a third specialist. This third specialist interpret the signal of OCTA at the polyps where the 2 specialists agreed on. | This was further clarified in line 85, and line 105 |
| 3. Please clarity if remission’ is regarding to the regression of polyp (i.e. change in size) and/or polyp activity (i.e. leakage)? | The term “remission” in the study referred to regression of the polyp in ICGA whether it was completely disappeared or decreased in size. This had been defined at the Materials & Methods, line 90. The decreased activity with decreased subretinal fluid or no fluid was not regarded as remission. The activity change, e.g., SRF, PED, had been described in Table 1. However, considering that regression was more commonly used and to avoid misunderstanding, we had revised by replacing “remission” with “regression”. Thank you. | The “remission” and been revised to “regression” in the text in line 29, 35, 36, 90, 91, 170, 176, 183, 190, 199, 201, 205, 219, 221, 229, 234, 247, 253. |
| 4. Please review any updated information on longer term PCV outcomes in the context of polyp remission. | A recent study with up to 11 years of follow-up showed that the long-term risk for massive submacular hemorrhage was significantly higher in patients with persistent polyps than patients without. (Chou et al, Retina 2020; 40:468-476.) | This long term FU study of PCV had been added and discussed in line 229. |
| Reviewer 3 |  |  |
| 1. Line 106 - µm should be used instead of um | Thank you for pointing out our error. | The correction had been made in page 6, line 114. |
| 2. Line 211 – PLANET study needs to be in caps. | Thank you for pointing out our error. | The correction had been made at page 12, line 234 |
| 3. Line 236 – spelling of polyps | Thank you for pointing out our error. | The correction had been made at page 13, line 264. |
| 4. Figure 2 – Lots of the boxes are blank needs to be clearly formatted | Figure 2 is the flow chart of all the outcomes of polyps. The blanks might be due to format converting error and was probably incompletely uploaded. We will re-submit and upload again and will check with the editor office to ensure the completeness of the figure. | Figure 2 would be re-submitted. |
| Case 2 in Table 1- mentions the location of the OCTA flow signal as Outer retina? That does not qualify for a PCV polyp OCTA flow signal and raises doubt as to whether it was a projected artefact. This is a critical error that needs to be corrected. In fact, the location of all OCTA PCV polyps needs to be beneath RPE (All the other polyps here ,except this, have that). Ideally, the depth of the signal below the RPE could be specified. If that is not possible this aspect of the table may be omitted altogether as it adds no useful information. | We apologize for such error. The OCTA of the case was reviewed, and confirmed that the polyp was still beneath the RPE. Thank you for pointing out our mistake. | Table 1 and page 8 line 158 had been revised. |
