# Supplementary material for: Flow signal change in polyps after anti-vascular endothelial growth factor therapy
Source: PLoS One. 2020 Oct 23;15(10):e0241230. doi: 10.1371/journal.pone.0241230 (PMC7584188; doi:10.1371/journal.pone.0241230)
Supplement: Attachment — Submitted filename: Response to Reviewers (second revision).docx [file pone.0241230.s002.docx]

Dear Editors-in-chief:

We appreciate the reviewers’ valuable questions and the chance to improve our manuscript. The concerns on the figure 1 had been explained and revised accordingly. We hoped after these revisions, the PLOS ONE will consider our paper interesting and deserve to be published.

Best regards,

Shih-Jen Chen MD, PhD

| Comments from reviewers | Response | Changes made in the manuscript |
| --- | --- | --- |
| Reviewer 1 |  |  |
| 1. Figure 1 (F, G, H) images are not showing the segmentation line. The en-face OCTA scans do not show any part of vasculature. Even the adjacent high flow polyp is not showing flow signal on en-face OCTA. Can the authors provide any other representative ICGA and OCTA images of low flow polyp? | We thank the reviewer’s comments and suggestion. We had revised and provide the en-face image in figure 1 (F,G,H). The revised segmentation line with widened slab allow us to visualize the high flow and low flow polyps on en-face OCTA even they are at different height beneath the RPE with prominent subretinal fluid above. The 3 consecutive horizontal scan of the low flow polyp showed no flow signal comparing to the adjacent high flow polyp.  The figure legend of figure 1 were also revised accordingly. | Figure 1 was revised as recommended.  Figure 1 legends:  **ICGA and OCTA of high-flow polyp and low-flow polyp.** (A) ICGA revealed one polyp (arrow). (B~D) The polyp (arrow) was identified by en face flow OCTA, and flow signal (arrow) was detectable in all 3 consecutive horizontal scans at the center of the polyp. The polyp was then defined as “high flow.” (E) Two polyps identified on ICGA (arrow and dotted arrow). (F~H) En face OCTA and 3 consecutive horizontal scan of the 2 types of polyps. The flow signal of “high flow” polyp (arrow) was detectable in en face and all 3 consecutive horizontal scans, while the flow signal of the “low flow”polyp (dotted arrow) could not be detected in en face and all 3 horizontal scans. |
